# Supplementary material for: Direct experimental observation of the molecular Jeff = 3/2 ground state in the lacunar spinel GaTa4Se8
Source: Nat Commun. 2017 Oct 4;8:782. doi: 10.1038/s41467-017-00841-9 (PMC5627251; doi:10.1038/s41467-017-00841-9)
Supplement: Supplementary file 1 — Supplementary Information [file 41467_2017_841_MOESM1_ESM.pdf]

Description of Supplementary Files

File Name: Supplementary Information
Description: Supplementary Figures, Supplementary Notes and Supplementary References

File Name: Peer Review File
Description:

Supplementary Figures

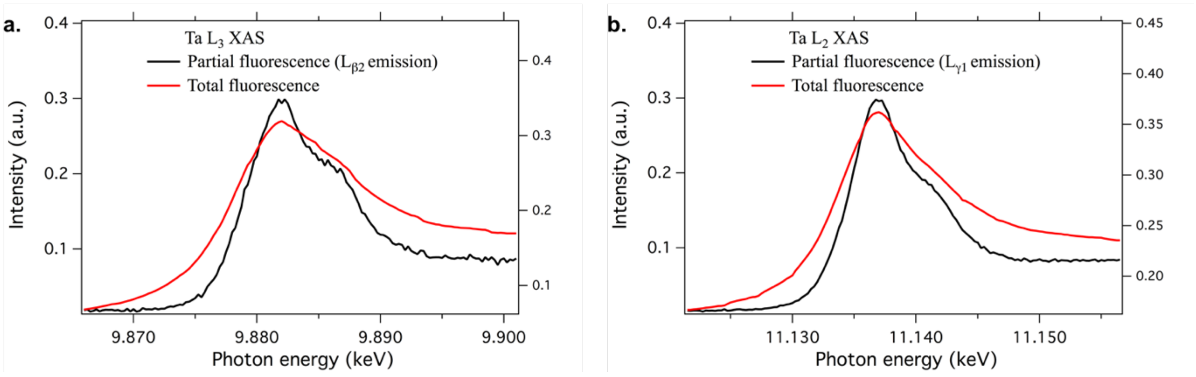

Supplementary Figure 1| Comparison of partial yield and total yield XAS spectra.

In both the L_3 and L_2 edges, the partial fluorescence x-ray absorption spectroscopy (XAS) spectra show much sharper absorption peaks than the conventional total yield XAS spectra because of a longer lifetime of the final states ($4d^{5/2}$ core-hole in the $L_{\beta2}$ emission and $4d^{3/2}$ core-hole in the $L_{\gamma1}$ emission).

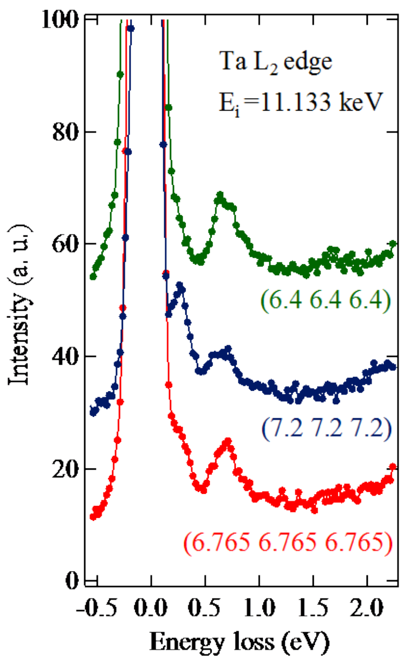

Supplementary Figure 2| L_2-edge RIXS spectra at scattering angles lower than 90°.

The Ta L_2-edge energy is 1.254 keV higher than the Ta L_3-edge energy. Therefore, the momentum transfers (**Q**) of the L_2-edge resonant inelastic x-ray scattering (RIXS) spectra (Fig. 3b) are higher than those of the L_3-edge RIXS spectra (Fig. 3a). For direct comparison, the L_2-edge RIXS spectra were measured at the lower **Q** values than the L_3-edge RIXS spectra in Fig. 3a. Three representative L_2-edge RIXS spectra are plotted for different **Q** values indicated. Because of much lower scattering angles than 90°, the elastic scattering (Thomson scattering) peak becomes significant. Two peaks at the 0.27 and 0.7 eV energy loss positions are clearly observed for all **Q** values. However, no peak structure exists in the 1.3 eV energy loss region for all measured **Q** values.

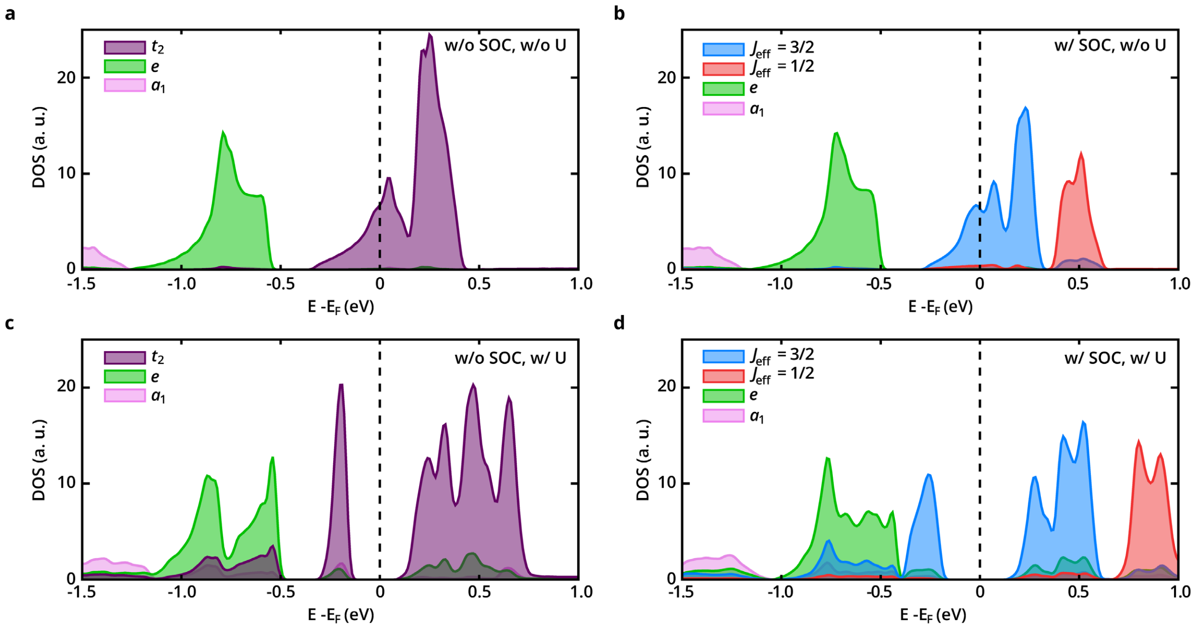

Supplementary Figure 3| Calculated DOS of $GaTa_4Se_8$ with and without a SOC and electron correlation *U*. Density of states (DOS) (**a**) without (w/o) a spin-orbit coupling (SOC) and Coulomb repulsion (*U*), (**b**) with (w/) a SOC and w/o *U*, (**c**) w/o a SOC and w/ *U*, and (**d**) w/ a SOC and *U*. These figures correspond to the schematic figure in Fig. 4a in the main text. Blue, red, violet, green and pink colors represent the $J_{\text{eff}} = 3/2$ and $J_{\text{eff}} = 1/2$, t_2, *e*,

a_1 MO characters, respectively. The vertical dashed lines represent the Fermi level E_F. 3eV of U is used for **c** and **d**.

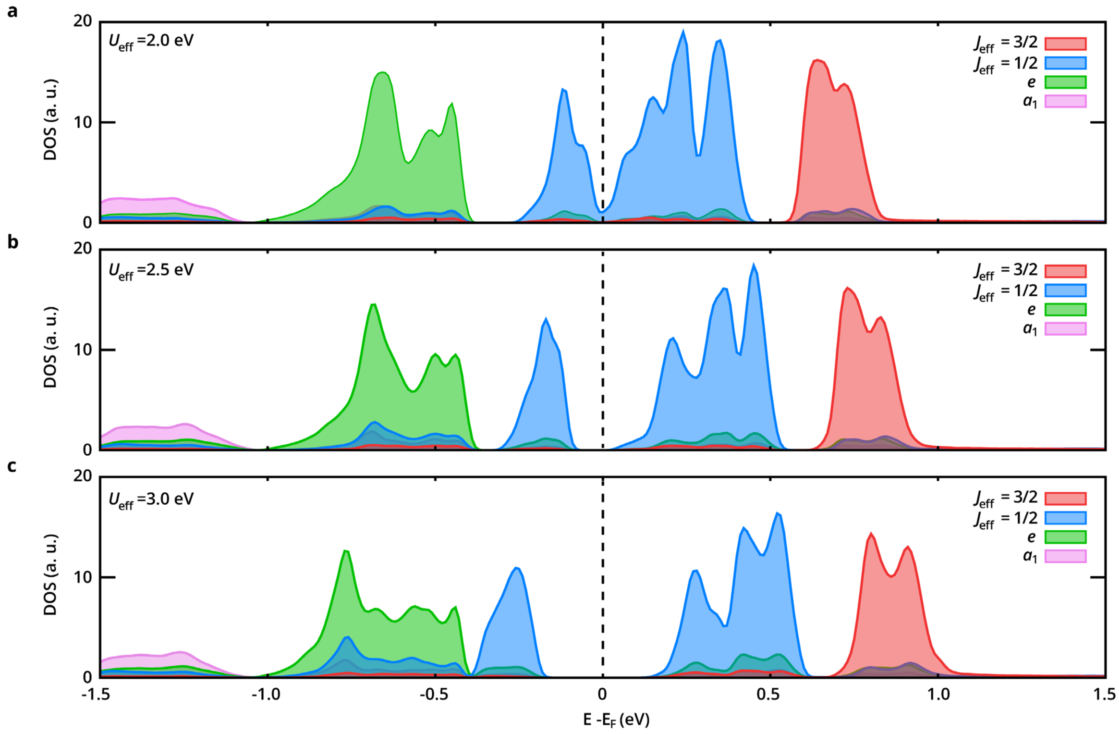

Supplementary Figure 4| U_{eff} dependence of DOS. DOS for (**a**) U_{eff} = 2 eV, (**b**) U_{eff} = 2.5 eV, and (**c**) U_{eff} = 3 eV. Blue, red, green and pink colors represent the J_{eff} = 3/2, J_{eff} = 1/2, e and a_1 MO characters, respectively. The vertical dashed lines respect the E_F.

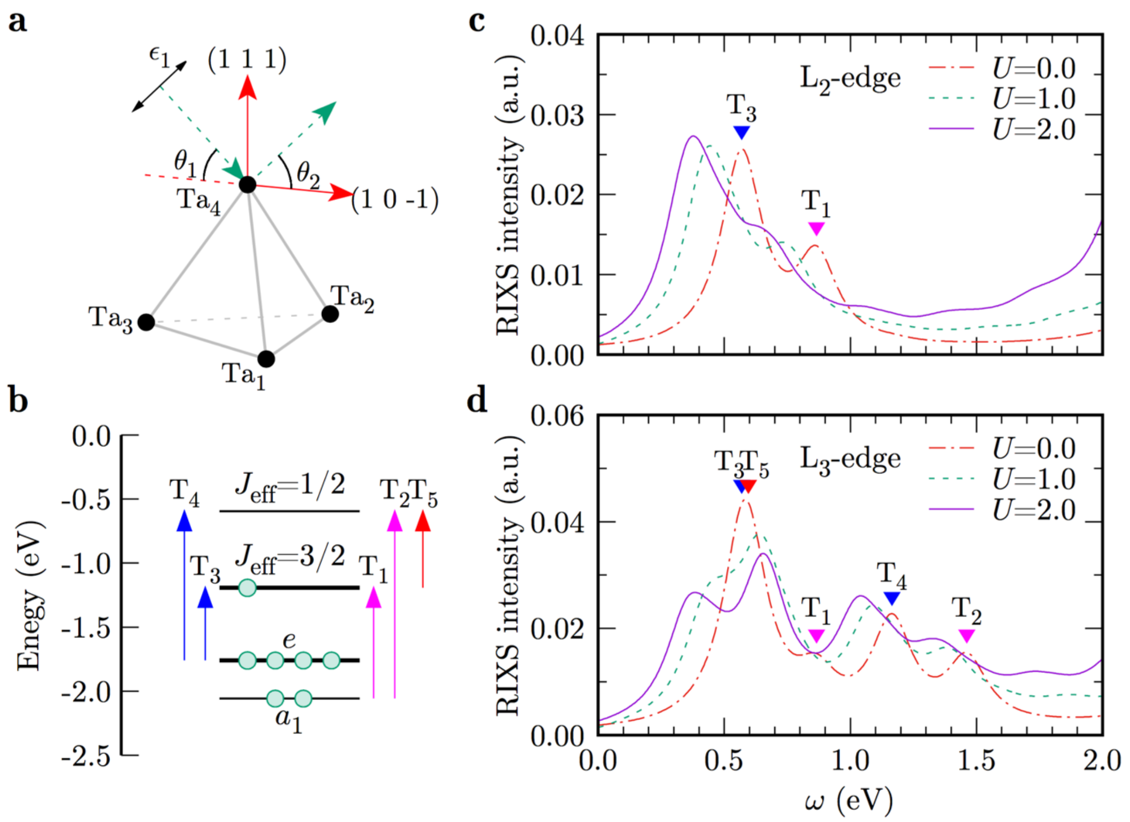

Supplementary Figure 5| Cluster model calculations of RIXS spectra. (**a**) Schematic diagram of a four-site tetrahedron cluster (Ta_1, Ta_2, Ta_3, and Ta_4) and the geometry of incident and outgoing x-rays. In the calculations, we set $\theta_1 = \theta_2 = 45°$ and an incident x-ray has π-polarization. (**b**) Energy diagram of four lowest MO states (a_1, e, $J_{\rm eff}$=3/2, and $J_{\rm eff}$=1/2) in the non-interacting limit with $t_\sigma = -1.41$ eV, $t_\pi = 0.10$ eV, $t_\delta = 0.213$ eV, and $\lambda_{\rm SO} = 0.4$ eV. The MO states are composed of four sets of Ta t_{2g} orbitals in the four-site tetrahedron cluster. The cluster contains seven electrons and hence the a_1 and e MO states are fully occupied, the $J_{\rm eff}$=3/2 MO states are partially occupied, and the $J_{\rm eff}$=1/2 MO state are fully unoccupied. Possible interband transitions ($\rm T_1$, $\rm T_2$, …, $\rm T_5$) are also indicated with arrows. (**c, d**) L_2- and L_3-edge RIXS spectra calculated for $U = 0$, 1, and 2 eV. Triangles indicate excitations corresponding to interband transitions $\rm T_1$-$\rm T_5$ in (**b**). For clarity, the elastic contributions are not shown in (c) and (d). Because the $J_{\rm eff}$=1/2 MO states comprise

mostly the Ta atomic j=5/2 states, the L_2-edge RIXS excitations involving the J_{eff}=1/2 MO states are largely suppressed. In contrast, the a_1, e, and J_{eff}=3/2 MO states are branched off from both the Ta atomic j=3/2 and 5/2 states (see Fig. 4b), and therefore these states can involve the dipole transitions to/from Ta $2p^{1/2}$ as well as $2p^{3/2}$.

Supplementary Notes

Supplementary Note 1| The interaction parameter *U* for DFT calculation. The value of U used in DFT calculation can be a delicate issue. Our calculations show that the gap is opened with U_{eff} ($\equiv U - J$) = 2.1 eV, and it keep increasing as U_{eff} increases. While the best comparison with the transport gap might be found at $U_{eff} \cong 2.8$ eV [1], we take U_{eff} = 2.3 eV for our main data. Any of our conclusions is not affected by this choice. The reasonable range is roughly 2.1 eV$<U_{eff}<$3.0 eV, which is quite consistent with the previous studies. For example, U = 2.27 eV has been used for TaS_2 based on the linear response calculation[2,3]. When the same technique is applied to $GaTa_4Se_8$, we obtain 2.33 eV. The previous cRPA (constrained random phase approximation) calculations[4] of 5d materials estimate U_{eff} ~2.0, ~1.6, ~2.0, ~1.8, and ~3.2 eV for elemental Ta[5], $NaOsO_3$[6], Sr_2IrO_4[7,8], Ba_2IrO_4[8] and Na_2IrO_3[9], respectively. These values could be slightly underestimated[10]. In the other studies for $MTaO_3$ (M=Ca, Sr, Ba)[11] and Ta-based perovskite oxides[12-14], U_{eff} =2 and $\geq$3 eV have been adopted.

Supplementary Note 2| Cluster model Hamiltonian. To calculate the RIXS spectra, we employed a three-band (i.e., t_{2g}-band) Hubbard model in a four-site tetrahedron cluster (see Supplementary Figure 5(a)) described by the following Hamiltonian:

$$H = \sum_{\langle i,j\rangle,\alpha,\beta,s} \left(h^{ji}_{\alpha\beta} c^\dagger_{j\alpha s} c_{i\beta s} + h.c. \right) + \lambda_{\mathrm{SO}} \sum_{i,\alpha,\beta,s,s'} (\mathbf{l} \cdot s)_{\alpha s,\beta s'} c^\dagger_{i\alpha s} c_{i\beta s'} + \frac{U}{2} \sum_{i,\alpha,\beta,s,s'} c^\dagger_{i\alpha s} c^\dagger_{i\beta s'} c_{i\beta s'} c_{i\alpha s}, \qquad (1)$$

where $c^\dagger_{i\alpha s}$ is the creation operator of electron with orbital α (=d_{xy}, d_{yz}, and d_{zx}) and spin s at site i, and $\mathbf{l}$ ($\mathbf{s}$) is the orbital (spin) angular momentum operator. We assumed that only three types of hopping channels (σ-, π-, and δ-type) between adjacent Ta atoms are accessible. For the nearest neighbor hoppings between Ta atoms in the *xy*-plane, such as Ta_1-Ta_2 and Ta_3-Ta_4 in Supplementary Figure 5a, $h^{12}_{xy,xy} = h^{34}_{xy,xy} = t_\sigma$, $h^{12}_{yz,yz} = h^{12}_{zx,zx} = h^{34}_{yz,yz} = h^{34}_{zx,zx} = t_\delta$, and $h^{12}_{yz,zx} = h^{12}_{zx,yz} = -h^{34}_{zx,yz} = -h^{34}_{yz,zx} = t_\pi$ can be non-zero. Other hopping parameters are also determined according to the tetragonal symmetry. The second and third terms of the Hamiltonian *H* describe the SOC and the on-site Coulomb repulsion, respectively. All parameters in *H* were set so that not only the energy level splitting of the MO states is consistent with the band structure calculations but also the experimental RIXS spectra are well fitted. The parameters used are $t_\sigma = -1.41$ eV, $t_\pi = 0.10$ eV, $t_\delta = 0.213$ eV, $\lambda_{\mathrm{SO}} = 0.4$ eV, and $U = 2.0$ eV. The total number of electrons is set to be seven.

Supplementary Note 3| Cluster model calculations of RIXS spectra. Let us first assume that the x-ray incomes with the energy $\omega_{\mathbf{k}_1}$, momentum $\mathbf{k}_1$, and polarization ϵ_1, and outgoes with energy $\omega_{\mathbf{k}_2}$, momentum $\mathbf{k}_2$, and polarization ϵ_2. The inelastic x-ray scattering thus induces the energy and momentum transfer, $\omega = \omega_{\mathbf{k}_1} - \omega_{\mathbf{k}_2}$ and $\mathbf{Q} = \mathbf{k}_1 - \mathbf{k}_2$, respectively. In the limit of the fast collision approximation (zero-th order of ultra-short lifetime expansion) and with the dipole approximation, the RIXS intensity $I(\omega, \mathbf{Q}, \epsilon_1, \epsilon_2)$ can be given as the following continued fraction form[15]:

$$I(\omega, \mathbf{Q}, \epsilon_1, \epsilon_2) = -\frac{1}{\pi\Lambda}\mathrm{Im}\left[\left\langle \Psi_g \middle| R(\epsilon_2, \epsilon_1, \mathbf{Q}) \frac{1}{\omega - H + E_g + i\delta} R(\epsilon_2, \epsilon_1, \mathbf{Q}) \middle| \Psi_g \right\rangle\right] \quad (2)$$

where $|\Psi_g\rangle$ is the ground state with its energy E_g and Λ is the x-ray broadening. δ is the Lorentz broadening of spectrum. In calculation, we set δ=0.1 eV. The RIXS scattering operator $R(\epsilon_2, \epsilon_1, \mathbf{Q})$ is given as

$$R(\epsilon_2, \epsilon_1, \mathbf{Q}) = \sum_i \sum_{\alpha\beta s} e^{i\mathbf{Q}\cdot\boldsymbol{r}_i} T_{\beta\alpha}(\epsilon_2, \epsilon_1) c_{i\beta s} c^{\dagger}_{i\alpha s} \quad (3)$$

where $T_{\beta\alpha}(\epsilon_2, \epsilon_1) = \sum_p \langle\varphi_p|\epsilon_2 \cdot \mathbf{r}|\varphi_\beta\rangle\langle\varphi_\alpha|\epsilon_1 \cdot \mathbf{r}|\varphi_p\rangle$ and $\mathbf{r}$ is a position operator. For L_2- and L_3-edge RIXS processes, the core-level φ_p summation is done over all wave functions of 2*p* *j*=1/2 and *j*=3/2 states. In order to mimic the experimental setup, we considered the x-ray geometry shown in Supplementary Figure 5a. We assumed the incident x-ray has π-polarization, whereas the outgoing x-ray has arbitrary polarization. We also set $\theta_1 = \theta_2 = 45°$. Note that $\mathbf{Q}$ should be parallel to (111) axis in the geometry**.** Since phase terms of three Ta sites (Ta_1, Ta_2, Ta_3) in $R(\epsilon_2, \epsilon_1, \mathbf{Q})$ are always the same, we only considered two cases in which $\left(e^{i\boldsymbol{Q}\cdot\boldsymbol{r}_1}, e^{i\boldsymbol{Q}\cdot\boldsymbol{r}_2}, e^{i\boldsymbol{Q}\cdot\boldsymbol{r}_3}, e^{i\boldsymbol{Q}\cdot\boldsymbol{r}_4}\right)$ is $(+1, +1, +1, +1)$ and $(+1, +1, +1, -1)$. The results shown in Fig. 5b in the main text and Supplementary Figs. 5c and 5d are obtained by averaging the calculations for these two different phase factors because the experimental setup for **Q** is somewhere between these two cases. For clarity, the elastic contribution is not shown in the calculated RIXS spectra.

Supplementary Note 4| Identification of L_2- and L_3-edge RIXS excitations.

Supplementary Figs. 5c and 5d show the calculated L_2- and L_3-edge RIXS spectra for $U = 0$, 1, and 2 eV. When $U = 0$, the RIXS peaks can be identified in terms of the interband transitions among the non-interacting MO states. As shown in Supplementary Figure 5b, five

types of transitions (T_1-T_5) can be possible among the four lowest MO states when seven electrons are considered, i.e., T_1: transition between a_1 and $J_{\text{eff}} = 3/2$ MO states, T_2: transition between a_1 and $J_{\text{eff}} = 1/2$ MO states, T_3: transition between e and $J_{\text{eff}} = 3/2$ MO states, T_4: transition between e and $J_{\text{eff}} = 1/2$ MO states, and T_5: transition between $J_{\text{eff}} = 3/2$ and $J_{\text{eff}} = 1/2$ MO states. We can easily identify that the lowest two excitations of L_2-edge RIXS spectrum for $U = 0$ are exactly due to transitions T_1 and T_3, while other excitations are almost inert. In case of L_3-edge RIXS spectrum, however, all five transitions T_1-T_5 are clearly manifested even though the excitations corresponding to transitions T_3 and T_5 are almost coincident in our selected parameters. When a finite U is introduced, the MO picture is somewhat disturbed due to the on-site Coulomb repulsion. However, we have still clearly observed in Supplementary Figures 5c and 5d that the excitations corresponding to transitions T_1-T_4 are a little shifted downward in energy with increasing U, whereas the excitation corresponding to transition T_5 is shifted upward. When U=2 eV, the excitation corresponding to transition T_3 appears at ~0.3 eV, the excitations corresponding to transitions T_1 and T_5 are located at almost the same energy around 0.7 eV, and the excitations originating from transitions T_2 and T_4 give rise to broaden peak structure at ~1.3 eV.

Supplementary References

1. Guiot, V., Janod, E., Corraze, B. & Cario, L. Control of the electronic properties and resistive switching in the new series of Mott insulators GaTa4Se8-yTey (0≤y≤6.5). *Chem. Mater.* **23**, 2611-2618 (2011).
2. Cococcioni, M. & de Gironcoli, S. Linear response approach to the calculation of the effective interaction parameters in the LDA + U method. *Phys. Rev. B* **71**, 035105 (2005)
3. Darancet, P., Millis, A. J., & Marianetti, C. A. Three-dimensional metallic and two-dimensional insulating behavior in octahedral tantalum dichalcogenides. *Phys. Rev. B* **90**, 045134 (2014).
4. Aryasetian, F. *et al.* Frequency-dependent local interactions and low-energy effective model from electronic structure calculations. *Phys. Rev. B* **70**, 195104 (2004)
5. Şaşıoğlu, E., Friedrich, C. & Blügel, S. Effective Coulomb interaction in transition metals from constrained random-phase approximation. *Phys. Rev. B* **83**, 121101(R) (2011).
6. Kim, B. *et al.* Lifshitz transition driven by spin fluctuations and spin-orbit renormalization in $NaOsO_3$. *Phys. Rev. B* **94**, 241113(R) (2016).
7. Martins, C., Aichhorn, M., Vaugier, L. & Biermann, S. Reduced Effective Spin-Orbital Degeneracy and Spin-Orbital Ordering in Paramagnetic Transition-Metal Oxides: Sr_2IrO_4 versus Sr_2RhO_4. *Phys. Rev. Lett.* **107**, 266404 (2011).
8. Arita, R., Kuneš, J., Kozhvnikov, A. V., Eguiluz, A. G. & Imada, M. *Ab initio* Studies on the Interplay between Spin-Orbit Interaction and Coulomb Correlation Sr_2IrO_4 versus Ba_2IrO_4. *Phys. Rev. Lett.* **108**, 086403 (2012).
9. Kim, M., Kim, B. H. & Min, B. I. Insulating Nature of Na_2IrO_3: Mott-type or Slater-type. *Phys. Rev. B* **93**, 195135 (2016).

10. Casula, M. et al. Low-energy models for correlated materials: bandwidth renormalization from coulomb screening. *Phys. Rev. Lett.* **109**, 126408 (2012).

11. Ali, Z., Khan, I., Ahmad, I., Khan, M. S. & Asadabadi, S. J. Theoretical studies of the paramagnetic perovskites MTaO3 (M = Ca, Sr and Ba). *Mater. Chem. Phys.* **162**, 308-315 (2015)

12. Khandy, S. A., & Gupta, C. D. Structural, elastic and thermo-electronic properties of paramagnetic perovskite $PbTaO_3$. *RSC. Adv. B* **6**, 48009-48015 (2016).

13. Oja *et al.* d^0 Ferromagnetic Interface between Nonmagnetic Perovskites. *Phys. Rev. Lett.* **109**, 127207 (2012).

14. Cooper, V. R. Enhanced carrier mobilities in two-dimensional electron gases at III-III/I-V oxide heterostructure interfaces. *Phys. Rev. B* **85**, 235109 (2012).

15. Kim, B. H., Shirakawa, T. & Yunoki, S. From a quasimolecular band insulator to a relativistic Mott insulator in t_{2g}^5 systems with a honeycomb lattice structure. *Phys. Rev. Lett.* **117**, 187201 (2016)
